# First report of the genus *Cratera* (Platyhelminthes, Geoplanidae) in Argentina, with description of a new species and comments on the species of the genus

**DOI:** 10.3897/zookeys.610.9465

**Published:** 2016-08-11

**Authors:** Lisandro Negrete, Francisco Brusa

**Affiliations:** 1 Universidad Nacional de La Plata, CONICET, FCNyM, Boulevard 120 & 61, B1900FWA, La Plata, Buenos Aires, Argentina

**Keywords:** Cratera viridimaculata sp. n., land planarians, Geoplaninae, Argentinian Atlantic Forest

## Abstract

A new species of land planarians of the genus *Cratera* is described. *Cratera viridimaculata* **sp. n.** was recorded in the Atlantic Forest Ecoregion, in north-eastern Argentina, and represents the first report of the genus *Cratera* outside Brazil. The new species is about 50 mm in length and externally characterized by a color pattern consisting of a light green olive pigmentation on the dorsum, stippled with dark gray fine spots, and dorsal eyes. Other features regarding the internal anatomy are the presence of a glandular margin, Cutaneous Muscular Index (CMI) of 10–13%, pharynx cylindrical, prostatic vesicle extrabulbar, tubular and C-shaped, with a proximal bifurcated portion, penis papilla protrusible with ejaculatory duct widened in its distal portion, and female atrium funnel-shaped. The new species is compared and discussed with its congeners.

Cutaneous Muscular Index

## Introduction

The genus *Cratera* Carbayo et al., 2013 is one of the currently recognized genera of land flatworms of the subfamily Geoplaninae. It is characterized by a peculiarity of the male reproductive system which is a widening of the distal part of the ejaculatory duct that traverses the penis papilla, reminiscent of a volcano crater in sagittal section, hence the origin of its generic name. Other features of the genus include prostatic vesicle extrabulbar; male atrium not folded and not separated from the female atrium; common ovovitelline duct dorsal to the female atrium; and genital canal dorso-anteriorly flexed, opening dorsally in the posterior region of the female atrium (Carbayo et al. 2013). *Cratera* was erected to separate some Brazilian species formerly described into the genus *Geoplana* Stimpson, 1857 as they share the features above mentioned. Five species were transferred by Carbayo et al. (2013) to this new genus, namely *Cratera crioula* (Froehlich, 1955), *Cratera joia* (Froehlich, 1956), *Cratera pseudovaginuloides* (Riester, 1938), *Cratera tamoia* (Froehlich, 1955), and *Cratera yara* (Froehlich, 1955). Since then, four new species of *Cratera* have been described, all of them recorded in the Brazilian Atlantic Forest (Rossi et al. 2014; Carbayo and Almeida 2015; Rossi et al. 2016). The Atlantic Forest extends along the Atlantic coast of Brazil and inland in this country, eastern Paraguay and north-eastern Argentina, where it is known as Interior Atlantic Forest, being characterized by semi-deciduous diversified forests. Even though the original coverage of the Atlantic forest has decreased significantly by human activities, this ecoregion still exhibits a high diversity, including land flatworms (Sluys 1998; Galindo-Leal and Câmara 2003). Here, we describe a new species of *Cratera* from the Interior Atlantic Forest of Argentina, the first record of this genus outside Brazil, extending its geographic range.

## Methods

Specimens were manually collected during the day below fallen logs in two natural reserves from north-eastern Argentina (Misiones Province), both located in the southern portion of the Interior Atlantic Forest ecoregion: Esmeralda Provincial Park (26°53'S, 53°52'W) and San Antonio Strict Nature Reserve (26°03'S, 53°46'W).

The animals were photographed alive and their external morphology was recorded. Then, they were killed with boiling water, fixed in 10% formaldehyde and subsequently conserved in 70% ethanol. Body fragments of land flatworms were dehydrated in an ascending series of ethanol, cleared in n-Butanol, embedded in Paraplast®, and serially sectioned with a microtome. Slides were stained with Masson’s trichrome method (Subarna et al. 2013). Type material was deposited in the Invertebrate Collection at Museo de La Plata
(MLP), Argentina.

## Results

### Order Tricladida Lang, 1884 Suborder Continenticola Carranza et al., 1998 Family Geoplanidae Stimpson, 1857  Subfamily Geoplaninae Stimpson, 1857 Genus *Cratera* Carbayo et al., 2013

#### Cratera viridimaculata sp. n.

Taxon classificationAnimaliaSeriataGeoplanidae

http://zoobank.org/A7CBCE5C-E265-46C3-83C7-F73DF0789675

**Figure 1.**
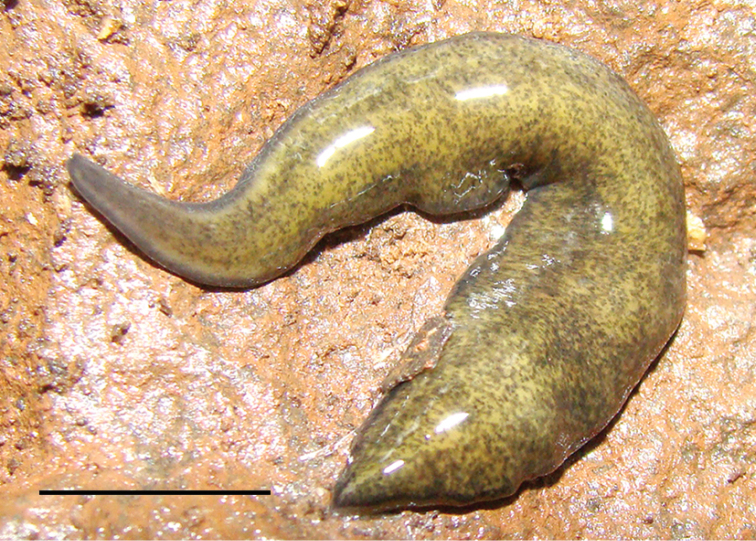
Dorsal view of a live specimen of *Cratera viridimaculata* sp. n. (holotype) (anterior end to the left). Scale bar: 10 mm.

**Figure 2.**
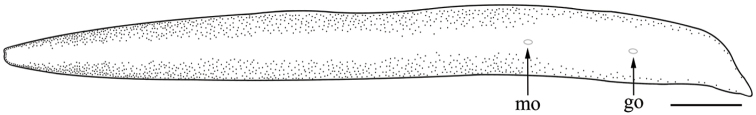
Schematic drawing of eyes pattern, in dorsal view, with position of mouth (**mo**) and gonopore (**go**) of *Cratera viridimaculata* sp. n. (paratype) (anterior end to the left). Scale bar: 5 mm.

**Figure 3.**
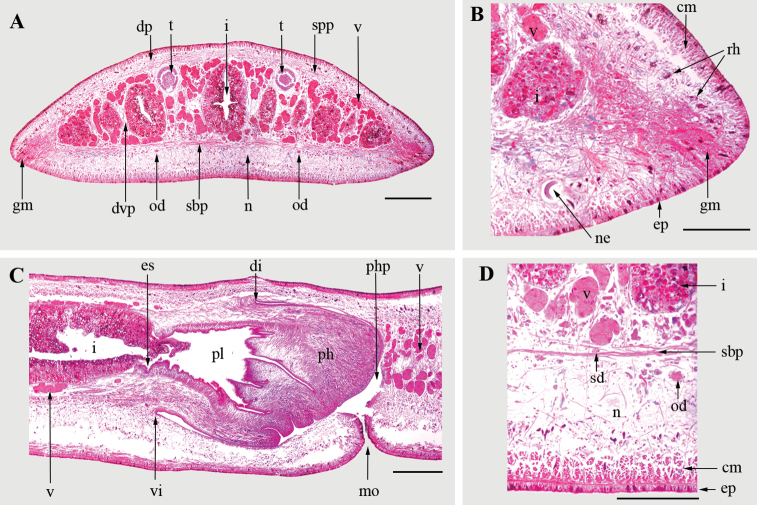
*Cratera viridimaculata* sp. n. (holotype). **A** Transverse section at pre-pharyngeal region **B** Detail of the body margin of a transverse section at pre-pharyngeal region **C** Sagittal section of the pharynx **D** Detail of a transverse section at pre-pharyngeal region. Abbreviations: **cm**, cutaneous musculature; **di**, dorsal insertion of pharynx; **dp**, dorsal parenchymatic musculature; **dvp**, dorsoventral parenchymatic fibers; **ep**, epidermis; **es**, esophagus; **gm**, glandular margin; **i**, intestine; **mo**, mouth; **n**, nervous plate; **ne**, nematode larva; **od**, ovovitelline duct; **ph**, pharynx; **pl**, pharyngeal lumen; **php**, pharyngeal pouch; **rh**, rhabditogen cells; **sbp**, sub-intestinal parenchymatic musculature; **sd**, sperm duct; **spp**, supra-intestinal parenchymatic musculature; **t**, testes; **v**, vitellaria; **vi**, ventral insertion of pharynx. Scale bars: 500 µm (**A**, **C**), 200 µm (**B**, **D**).

**Figure 4.**
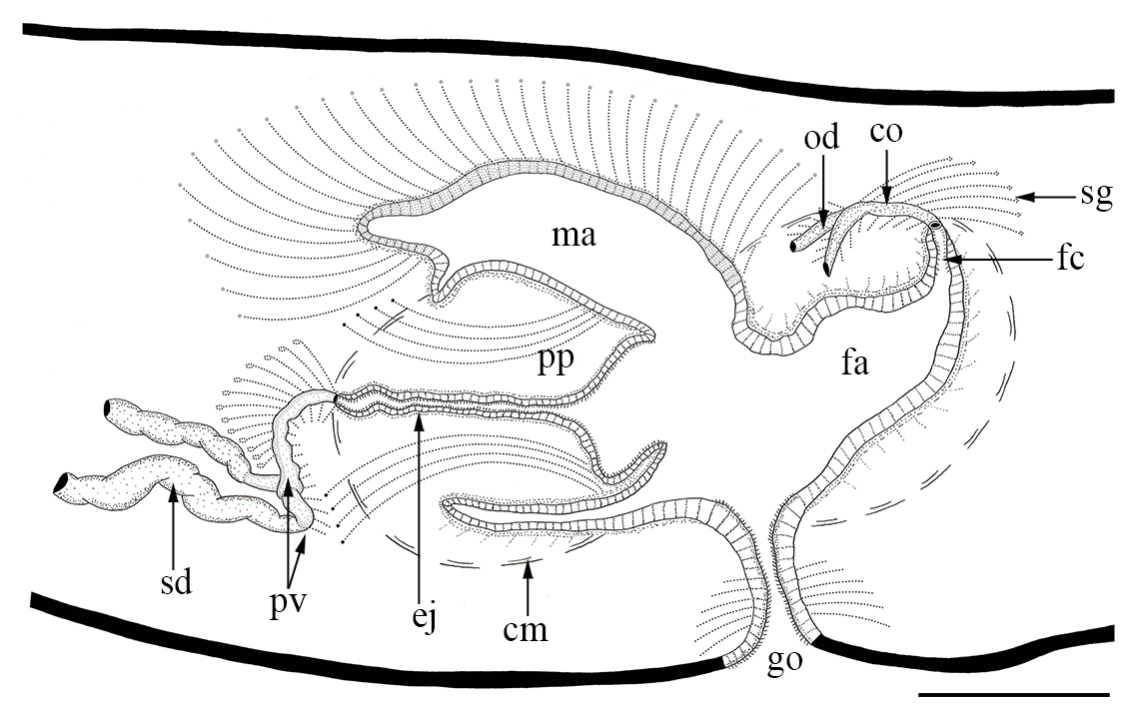
Schematic reconstruction, in sagittal view, of the copulatory apparatus of *Cratera viridimaculata* sp. n. (holotype). Abbreviations: **cm**, common muscle coat; **co**, common ovovitelline duct; **ej**, ejaculatory duct; **fa**, female atrium; **fc**, female genital canal; **go**, gonopore; **ma**, male atrium; **od**, ovovitelline duct; **pp**, penis papilla; **pv**, prostatic vesicle; **sd**, sperm duct; **sg**, shell glands. Scale bar: 500 µm.

**Figure 5.**
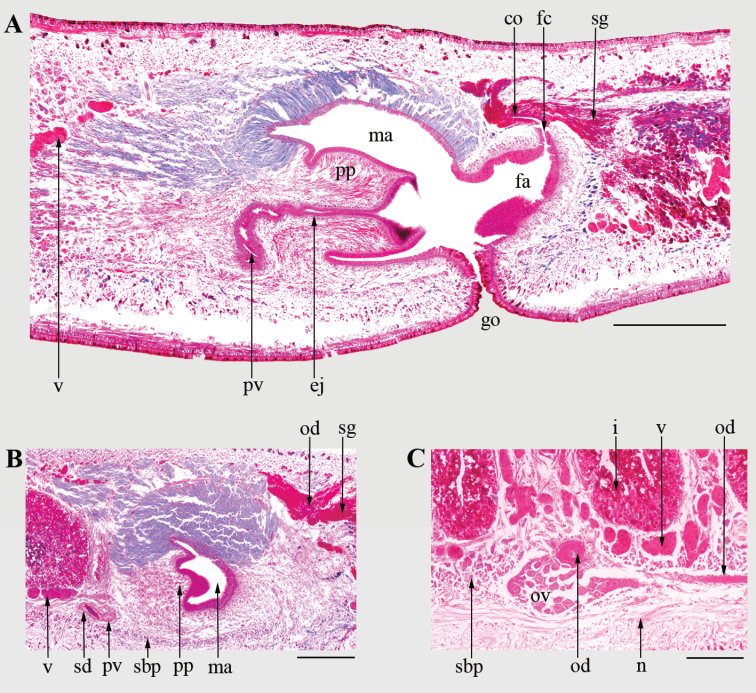
*Cratera viridimaculata* sp. n. (holotype). **A**, **B** Sagittal sections of the copulatory apparatus **C** Sagittal section of the anterior region, at the level of ovaries. Abbreviations: **co**, common ovovitelline duct; **ej**, ejaculatory duct; **fa**, female atrium; **fc**, female genital canal; **go**, gonopore; **i**, intestine; **ma**, male atrium; **n**, nervous plate; **od**, ovovitelline duct; **ov**, ovary; **pp**, penis papilla; **pv**, prostatic vesicle; **sbp**, sub-intestinal parenchymatic musculature; **sd**, sperm duct; **sg**, shell glands; **v**, vitellaria. Scale bars: 500 µm (**A**), 250 µm (**B**, **C**).

Figs 1 , 2 , 3 , 4 , 5 , Tables 1 , 2

Geoplana sp. 6 (Negrete et al., 2014 in part)

##### Type material.

**Holotype** (Figs 1, 3–5). MLP–He 6944. Locality: Esmeralda Provincial Park (26°53'S, 53°52'W), Misiones Province, Argentina. 19 June 2013; cephalic region: transversal sections on 16 slides (6 µm thick); anterior region: sagittal sections on 30 slides (7 µm thick); anterior region at level of ovaries: sagittal sections on 20 slides (7 µm thick); pre-pharyngeal region: transverse sections on 6 slides (6 µm thick); pharynx: sagittal sections on 32 slides (7 µm thick); copulatory apparatus: sagittal sections on 32 slides (7 µm thick).

**Paratype** (Fig. 2). MLP–He 6489. Locality: San Antonio Strict Nature Reserve (26°03'S, 53°46'W), Misiones Province, Argentina. 30 October 2008; cephalic region and anterior region at level of ovaries: sagittal sections on 28 slides (8 µm thick); pre-pharyngeal region: transverse sections on 12 slides (8 µm thick); pharynx: sagittal sections on 31 slides (8 µm thick); copulatory apparatus: sagittal sections on 31 slides (8 µm thick).

##### Type locality.

Esmeralda Provincial Park (26°53'S, 53°52'W), in native subtropical forest. Misiones province, Argentina.

##### Diagnosis.

Species of *Cratera* of 50 mm in length; dorsal surface stippled with dark gray fine spots on a light olive green background; eyes dorsal; glandular margin present; CMI, 10–13%; pharynx cylindrical; prostatic vesicle extrabulbar, tubular and C-shaped, with proximal bifurcated portion.

##### Description.

**External morphology.** Body elongate with parallel margins. Anterior tip blunt and posterior end pointed (Figs 1, 2). Dorsal surface light olive green, stippled with dark gray fine spots, and body margins and cephalic region pigmented dark gray (Fig. 1). Ventral surface whitish with margins grayish. After fixation, the dorsal color pattern became paler with lighter gray fine spots. Eyes distributed from the anterior tip to the posterior end. They surround the cephalic region and extend uniserially on body margins along 1–2 mm from the anterior tip, continuing pluriserially over the dorsal surface, being surrounded by clear halos. Eyes occupy about 30% of body width on each side of the dorsal surface at pre-pharyngeal region. Behind the pharynx, they decrease in number and at the level of the copulatory apparatus become uniserial and marginal (Fig. 2). After fixation, the length of specimens is about 50 mm, maximum width ~4.5 mm, and maximum height ~1.5 mm. Mouth and gonopore located at a distance of 66–75% and 83–89% from the anterior tip, respectively (Table 1).

**Table 1.** Measurements (mm) from fixed specimens of *Cratera viridimaculata* sp. n. CS, width of creeping sole; DG: distance from gonopore to anterior end; DM: distance from mouth to anterior end. The numbers given in parentheses represent the position relative to body length (%). Thickness (µm) of cutaneous (CM) and parenchymatic (PM) musculatures at pre-pharyngeal region. CMI (cutaneous muscular index): ratio between height of cutaneous musculature to body height. PMI (parenchymatic muscular index): ratio between height of parenchymatic musculature to body height. Both indices measured at pre-pharyngeal region. Abbreviations: cc, circular cutaneous musculature; dc, diagonal cutaneous musculature; dp, dorsal parenchymatic musculature; lc, longitudinal cutaneous musculature; sbp, sub-intestinal parenchymatic musculature; spp, supra-intestinal parenchymatic musculature.

Measurements	Holotype	Paratype	Measurements	Holotype	Paratype
**Length**	53	51	**CM dorsal (cc–dc–lc)**	2.5 – 10 – 45	2.5 – 10 – 50
**Width**	4.5	4.4	**CM ventral (cc–dc –lc)**	2.5 – 10 – 75	5 – 20 – 90
**Height**	1.5	1.4	**CMI**	10%	13%
**DM**	40 (75%)	33.8 (66%)	**PM (dp–spp–sbp)**	40 – 50 – 50	25 – 50 – 40
**DG**	47.3 (89%)	42.3 (83%)	**PMI**	9%	8%
**CS (%)**	90%	90%			

**Internal morphology.** Sensory pits, as simple invaginations ranging from 25 µm to 40 µm deep, contouring anterior tip and extending along body margins in a single irregular row. They occur at intervals of about 25–50 µm, and posteriorly become gradually spaced until they disappear at 5–6 mm from anterior tip. Three types of secretory cells discharge through dorsal epidermis (15 µm height) and body margins at pre-pharyngeal region: numerous rhabditogen cells with xanthophil secretion (rhammites), abundant cells with fine granular erythrophil secretion, and scarce cells with fine granular cyanophil secretion. Glandular margin composed of abundant fine granular erythrophil secretion and scarce fine granular xanthophil and cyanophil secretion (Fig. 3A, B). Ventral epidermis (25 µm height) ciliated on the creeping sole (90% of body width). Three types of secretory cells discharge their secretion through the creeping sole: rhabditogen cells (with rhabdithes), and abundant cells with fine granular erythrophil and cyanophil secretion. Cephalic region with the same types of secretory cells, discharging through dorsal and ventral epidermis but in less quantity, except cells with fine granular xanthophil secretion which are highly abundant mainly on body margins. No musculo-glandular specializations. Cutaneous musculature with the usual three layers present in the subfamily Geoplaninae: circular, oblique and longitudinal, the latter arranged in bundles and is the thickest (Table 1). Cutaneous Muscular Index (CMI) ranging from 10% to 13%. Parenchymatic musculature composed of a dorsal layer with oblique fibers, a supra-intestinal and a sub-intestinal transverse layers (Table 1) (Fig. 3A, B, D). Additionally, dorsoventral fibers located among intestinal branches (Fig. 3A). Parenchymatic Muscular Index (PMI) ranging from 8% to 9% (Table 1).

Pharynx cylindrical, 1.5–2.3 mm in length (3–4% of body length), with dorsal insertion located at the proximal third of pharyngeal pouch (3–3.2 mm in length) (Fig. 3C). Pharynx lined by ciliated cuboidal epithelium. Pharyngeal musculature of the planariid type comprising an outer musculature arranged in two layers: longitudinal subepithelial layer (5 µm thick) followed by a subjacent circular layer (5–10 µm thick). Pharyngeal lumen lined by ciliated columnar epithelium. Pharyngeal inner musculature comprised of circular subepithelial layer (75–90 µm thick) followed by a thinner longitudinal layer (10–20 µm thick). Pharyngeal glands constituted by three secretory cell types: abundant cells with fine granular erythrophil secretion, less abundant cells with fine granular cyanophil secretion and scarce cells with amorphous cyanophil secretion (Fig. 3C). Cell bodies of pharyngeal glands located in the surrounding parenchyma, mainly anterior to pharynx. Short esophagus (250–300 µm in length) lined by ciliated columnar epithelium, followed by a subepithelial circular layer (45–60 µm thick) and a subjacent longitudinal layer (5–15 µm thick). Esophagus: pharynx ratio, 13–17%.

Testes dorsal, mature, arranged in one irregular row on each side of the body, located between the supraintestinal parenchymatic muscle layer and intestinal branches (Fig. 3A). They extend immediately behind the ovaries to nearly the ventral root of pharynx (Table 2). Sperm ducts dorso-mediad to ovovitelline ducts, located among fibers of sub-intestinal transverse layer (Fig. 3D). Near the copulatory system, the lumen of sperm ducts is dilated and full of spermatozoa. They curve to the sagittal plane and communicate with the proximal paired portions of the prostatic vesicle (150–170 in length each) (Figs 4, 5A, B). Prostatic vesicle, extrabulbar, unpaired, tubular and C-shaped, spaced 5.2 mm from the pharyngeal pouch (Figs 4, 5A). Ejaculatory duct almost straight, except its proximal portion which is sinuous, opening through an expansion into the tip of the penis papilla (Figs 4, 5A). Male atrium with unfolded walls, housing a cylindrical penis papilla which occupies most of the atrium (Figs 4, 5A). Male atrium with ample communication with female atrium, without folds separating both atria (Figs 4, 5A).

**Table 2.** Measurements (mm) of reproductive organs of *Cratera viridimaculata* sp. n. DPVP, distance between prostatic vesicle and pharyngeal pouch; LCGD, length of common glandular ovovitelline duct; LFA, length of female atrium; LFC, length of female canal; LMA, length of male atrium; LPP, length of penis papilla; LPV, length of prostatic vesicle. The numbers given in parentheses represent the position relative to body length (%).

	Holotype	Paratype
**Anteriormost testes**	16 (30%)	12 (23%)
**Posteriormost testes**	37.1 (70%)	30.6 (60%)
**LPV**	0.5	0.45
**DPVP**	5.2	5.2
**LPP**	0.55	0.45
**LMA**	0.95	0.7
**Location of ovaries**	14 (26%)	11 (22%)
**LCGD**	0.25	0.15
**LFC**	0.1	0.1
**LFA**	0.65	0.45

Sperm ducts lined with ciliated cuboidal epithelium, coated by circular fibers (5 µm thick). Lining epithelium of prostatic vesicle columnar and ciliated, receiving abundant fine granular erythrophil secretion from glands with cells bodies located anterior to the prostatic vesicle. Muscularis of prostatic vesicle (15–20 µm thick) arranged in a circular layer interwoven with oblique fibers. Ejaculatory duct lined with ciliated columnar epithelium, which receives scarce fine granular erythrophil secretion, coated by circular fibers (2.5–5 µm thick). Penis papilla lined with non-ciliated columnar epithelium, strongly erythrophil (Fig. 5A). Epithelial lining of penis papilla receives abundant fine granular erythrophil secretion and less abundant amorphous erythrophil secretion (Fig. 5A). Cell bodies of penis glands located in the parenchyma, outside the penis bulb. Muscularis of the penis papilla (5–10 µm thick) composed of circular fibers. Male atrium lined with non-ciliated columnar epithelium, followed by circular muscle layer (5–15 µm thick). The epithelial lining of the dorsal wall of the male atrium receives large amount of fine granular cyanophil secretion, and less abundant fine granular erythrophil secretion (Fig. 5A, B). The ventral wall receives fine granular erythrohil secretion and scarce cyanophil granules. Cell bodies of glands which discharge their secretions into the male atrium located in the parenchyma, external to common muscle coat.

Ovaries ovoid and distally elongate, measuring 500–600 µm in length, located just below the sub-intestinal parenchymatic muscle layer (Fig. 5C). Ovovitelline ducts emerge dorso-laterally from the middle third of ovaries, and run posteriorly between sub-intestinal parenchymatic muscle layer and nerve plate (Figs 3A, D, 5C). At the level of gonopore, ovovitelline ducts ascend, run to the sagittal plane and join in a short common glandular ovovitelline duct (Figs 4, 5A, B). The common ovovitelline duct is horizontal and located above the posterior region of the female atrium (Figs 4, 5A). Short female genital canal dorsoventrally oriented, connecting common glandular duct and female atrium (Figs 4, 5A). Female atrium funnel-shaped and without folded walls, shorter than the male atrium (Figs 4, 5A, Table 2).

Ovovitelline ducts lined with ciliated cuboidal epithelium, coated by circular fibers (2.5 µm thick). Ascending portions of ovovitelline ducts receive secretion from shell glands (Fig. 5B). Lining epithelium of common glandular ovovitelline duct columnar and ciliated, receiving abundant secretion from shell glands and amorphous cyanophil secretion (Fig. 5A). Cell bodies of these glands located posterior to the copulatory apparatus (Figs 4, 5A). Female genital canal lined with ciliated columnar epithelium, coated by circular fibers (5–10 µm thick). Female atrium lined by non-ciliated columnar epithelium, with nuclei located at different heights and giving a stratified aspect (Fig. 5A). Muscularis of female atrium composed of circular fibers mixed with some longitudinal fibers (10–15 µm thick). Female genital canal and female atrium receive abundant fine granular erythrophil secretion, and fine granular cyanophil secretion in less quantity. Common muscle coat poorly organized, composed of longitudinal and oblique fibers (5–10 µm thick) (Fig. 4).

Vitellaria well-developed in both specimens studied, located among intestinal branches (Figs 3A–D, 5A–C). Gonopore canal slightly anteriorly flexed, lined with ciliated columnar epithelium (Fig. 5A). Three types of secretory cells discharge their secretion through the gonopore canal: rhabditogen cells (with rhabdithes), abundant cells with fine granular erythrophil secretion and scarce cells with fine granular cyanophil secretion.

##### Etymology.

The specific name refers to the dorsal pigmentation of body, stippled with dark gray dots on a light green olive background (from lat. *viridis* = green, greenish; *maculatus* = maculated, spotted, splattered with dots).

##### Distribution.

Southern portion of the Interior Atlantic Forest ecoregion, Misiones Province, north-eastern Argentina. The new species was found in native subtropical forests, in two natural reserves: Esmeralda Provincial Park (26°53'S, 53°52'W) and San Antonio Strict Nature Reserve (26°03'S, 53°46'W).

## Discussion

As with other genera of the subfamily Geoplaninae, the diagnosis of the genus *Cratera* Carbayo et al., 2013 relies on a combination of non-exclusive features, which mainly include medium-sized body, pharynx cylindrical to bell-shaped, prostatic vesicle extrabulbar, penis papilla protrusible, common ovovitelline duct and female canal dorsal to female atrium, and female atrium funnel-shaped (Carbayo et al. 2013). However, an autapomorphy of this genus is the presence of a widening of the ejaculatory duct in its opening in the apex of the penis papilla. This peculiarity and the other features above mentioned were observed in the new species herein described, strongly supporting its inclusion into this genus. The finding of a new species of *Cratera* outside Brazil extends the geographic range of this genus.

Taking into account the external morphology, among the species currently known of *Cratera*, the majority of them exhibit a well-defined stripe pattern on the dorsum, namely: *Cratera anamariae* Carbayo, 2015, *Cratera cuarassu* Carbayo & Almeida, 2015, *Cratera joia*, *Cratera pseudovaginuloides*, *Cratera steffeni* Rossi et al., 2014, *Cratera tamoia* and *Cratera yara*. Thus, they can be easily distinguished from *Cratera viridimaculata* sp. n. because the new species has scattered dots on the dorsal surface without forming stripe pattern. The remaining two species, *Cratera ochra* Rossi et al., 2016 and *Cratera crioula*, even though they have stripes, they also exhibit a stippled pattern on the dorsum. In *Cratera ochra* the dorsal color pattern is quite similar to that *Cratera viridimaculata* sp. n., with a yellow ochre pigment splashed with irregularly arranged grayish dots, except body margins which are free of dots. However, unlike the new species, in *Cratera ochra* the dots are concentrated forming two broad grayish bands (Rossi et al. 2016). Regarding *Cratera crioula*, this species can be distinguished from *Cratera viridimaculata* sp. n. because the stippled pattern follows a homogeneous arrangement on a dark gray background, only free of dots along of a thin median stripe and two para-marginal stripes, of whitish pigment (Froehlich 1955).

Regarding the copulatory apparatus, the new species shares with *Cratera anamariae*, *Cratera ochra*, *Cratera pseudovaginuloides*, *Cratera steffeni*, and *Cratera yara* the presence of highly abundant cyanophil secretion discharging onto the dorsal wall of the male atrium. Besides, *Cratera viridimaculata* sp. n. and the five species above mentioned have a tubular and sinuous extrabulbar prostatic vesicle, and similarly to *Cratera viridimaculata* sp. n., the prostatic vesicle of *Cratera anamariae*, *Cratera ochra* and *Cratera steffeni* has proximal bifurcated branches which connect with the sperm ducts. However, *Cratera viridimaculata* sp. n. differs from *Cratera ochra* and *Cratera steffeni* in the position of the prostatic vesicle. In these species, the proximal part of the unpaired portion is almost horizontal and dilated, with the bifurcated branches also expanded, giving a T shape (Rossi et al. 2014, 2016). In the new species, the unpaired portion is C-shaped and the proximal bifurcated branches are not expanded. In regard to *Cratera anamariae*, the unpaired portion of the prostatic vesicle is sinuous as in *Cratera viridimaculata* sp. n. but their paired proximal branches run dorso-anteriorly (Carbayo and Almeida 2015), while in the new species the bifurcated branches run almost horizontal in their course to the sagittal plane.

Some aspects about the internal anatomy of *Cratera crioula*, *Cratera cuarassu* and *Cratera joia* deserve comment. As previously noted, one of the most remarkable features of the genus *Cratera* is the presence of a widening of the ejaculatory duct in its distal portion. However, in the original descriptions of *Cratera crioula* and *Cratera joia* the authors did not mention this peculiarity, this being confirmed in the reconstructions of the copulatory apparatus (Froehlich 1955; Froehlich 1956). Moreover, in *Cratera joia* the prostatic vesicle is intrabulbar, in contrast to other species of *Cratera*, and the penis papilla extends beyond the gonopore and occupies half of the female atrium, which has not been observed in species of *Cratera*, and resembling more the species of the genus *Geoplana* Stimpson, 1857 (see Carbayo et al. 2013 for the emended diagnosis of *Geoplana*). However, the inclusion of *Cratera crioula* into the genus *Cratera* is supported by results of the molecular phylogeny of Geoplaninae accomplished by Carbayo et al. (2013), in which this genus was proposed. According these results, *Cratera crioula* form a clade with *Cratera tamoia*, *Cratera pseudovaginuloides*, *Cratera cuarassu* (= *Geoplana* sp. 5 in Carbayo et al. 2013), *Geoplana hina* Marcus, 1951, and some undescribed species. In contrast, *Geoplana hina* was not included in *Cratera* although molecular data appear to support it, but as in *Cratera joia*, the description of the anatomy of the copulatory apparatus (see Marcus 1951) seems to not fit with the diagnosis of the genus.

Regarding *Cratera cuarassu*, this species possesses a very short and wide penis papilla which hangs from the roof of the male atrium and occupies the entire atrium, whose proximal and distal walls have numerous folds (Carbayo and Almeida 2015). The peculiar shape of the penis papilla is distinguished from the other species of *Cratera*, in which the papilla is nearly horizontal, and even from any other species of Geoplaninae (Carbayo and Almeida 2015). Furthermore, Carbayo and Almeida (2015) have stated that *Cratera cuarassu* has a large intra-penial cavity as a result of the extension of the widening in the ejaculatory duct. Nevertheless, the inclusion of *Cratera cuarassu* into this genus is supported by molecular data (Carbayo et al. 2013), even though the copulatory apparatus exhibits numerous dissimilarities with the rest of species of *Cratera*, as male atrium folded and separated from the female one, female atrium without funnel-shape, in addition with the features about the penis papilla above mentioned.

In light of this morphological heterogeneity, it would be interesting to confirm the presence or absence of the distal widening of the ejaculatory duct in *Cratera crioula* and *Cratera joia* as well as a reanalysis of the internal anatomy as a whole. In regard to *Cratera joia*, some justification based on morphological or molecular data is missing, so its inclusion in *Cratera* is at least doubtful according to the anatomical features above mentioned. As in *Cratera crioula* and *Cratera joia*, a re-evaluation of the morphology of *Geoplana hina* could clarify this matter.

## Supplementary Material

XML Treatment for Cratera viridimaculata
